# Supplementary material for: Transsynaptic modulation of presynaptic short-term plasticity in hippocampal mossy fiber synapses
Source: Nat Commun. 2021 May 18;12:2912. doi: 10.1038/s41467-021-23153-5 (PMC8131630; doi:10.1038/s41467-021-23153-5)
Supplement: Supplementary file 1 — Supplementary Information [file 41467_2021_23153_MOESM1_ESM.pdf]

Transsynaptic modulation of presynaptic short-term plasticity in hippocampal mossy fiber synapses

David Vandael[1,2,3,✉], Yuji Okamoto[1,3], and Peter Jonas[1,✉]

1 Cellular Neuroscience, IST Austria (Institute of Science and Technology Austria), Am Campus 1, A-3400 Klosterneuburg, Austria

2 Present address: Netherlands Institute for Neuroscience, Royal Netherlands Academy for Arts and Sciences (KNAW), Meibergdreef 47, 1105 BA, Amsterdam, The Netherlands

3 These authors contributed equally.

✉ Corresponding authors. E-mail: david.vandael@ist.ac.at or peter.jonas@ist.ac.at

Corresponding authors:
Dr. David Vandael
Dr. Peter Jonas
IST Austria (Institute of Science and Technology Austria)
Cellular Neuroscience
Am Campus 1
A-3400 Klosterneuburg, Austria
Phone: 0043-2243-9000-3700
Fax: 0043-2243-9000-2007
E-mail: david.vandael@ist.ac.at
E-mail: peter.jonas@ist.ac.at

Supplementary Figure 1 | Pool analysis of PTP in 0.1 mM and 10 mM EGTA, as in Fig. 2a–c and Fig. 2d–f.

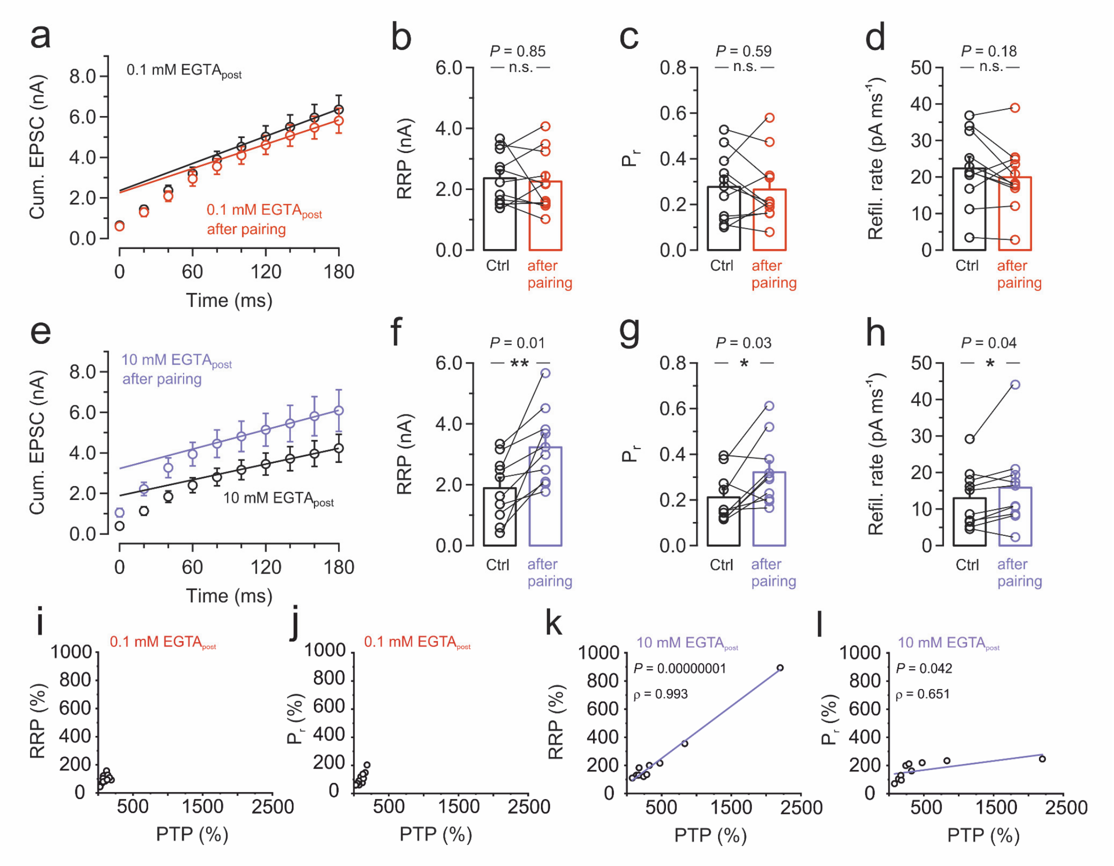

a–d, Pool analysis with 0.1 mM EGTA in the postsynaptic pipette. Last 4 data points were fit by linear regression, revealing readily releasable pool (RRP), release probability (P_r), and refilling rate.

e–h, Pool analysis with 10 mM EGTA in the postsynaptic pipette, inhibiting the suppression of PTP.

i–l, Scatter plot of increase in RRP (i, k) and P_r (j, l) against the magnitude of PTP for 0.1 mM EGTA (i, j) and 10 mM EGTA (k, l). For 10 mM EGTA, there is a highly significant correlation between RRP and PTP (Pearson's $\rho = 0.993$; $P < 0.001$), but only a moderately significant correlation between P_r and PTP (Pearson's $\rho = 0.651$; $P = 0.042$). * indicates $P < 0.05$, ** $P < 0.01$, and n.s. denotes non-significant difference ($P \geq 0.05$). Data in (a–d) are from 11 pairs, Data in (e–h) are from 10 pairs. Error bars indicate SEM.

Supplementary Figure 2 | Transsynaptic modulation of PTP at near-physiological temperature.

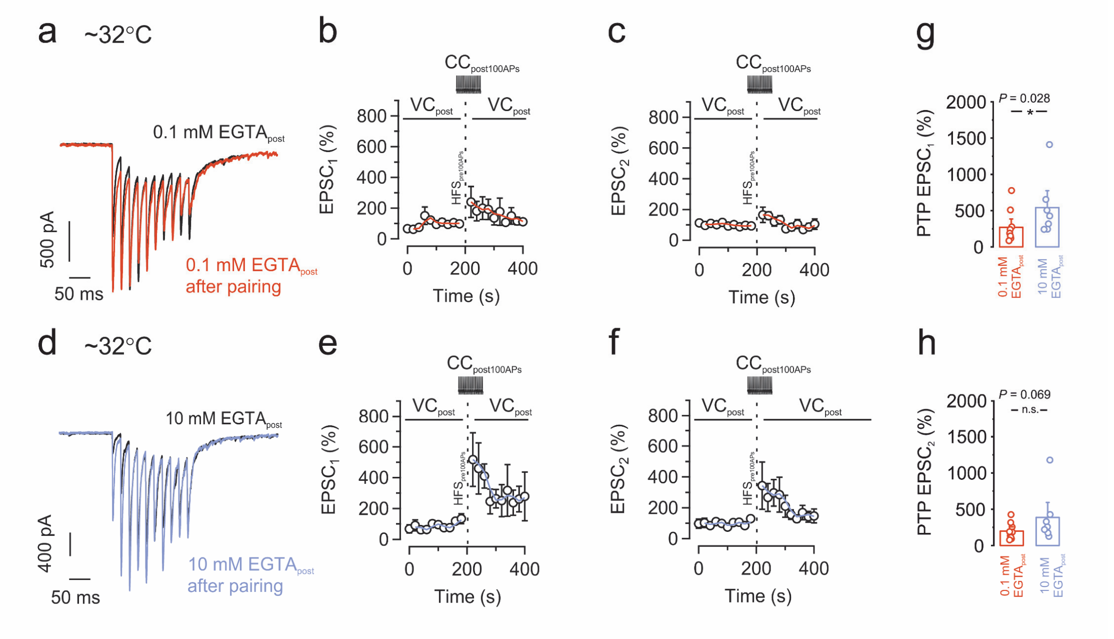

a, Representative traces of a 50 Hz train of EPSCs recorded at nearly physiological temperature before and after HFS_{100} pairing with 0.1 mM of EGTA in the postsynaptic pipette.

b,c, Plots of normalized $EPSC_1$ and $EPSC_2$ against experimental time at near-physiological temperature with 0.1 mM EGTA in the postsynaptic pipette.

d–f, Similar plots as in (a–c), but with 10 mM of EGTA in the postsynaptic pipette.

g,h, Summary bar graphs showing the percentage of PTP at near-physiological temperature after paired HFS_{100} stimulation with 0.1 mM EGTA and 10 mM EGTA in the postsynaptic pipette. Data for $EPSC_1$ (g) and $EPSC_2$ (h). * indicates $P < 0.05$, and n.s. denotes non-significant difference ($P \geq 0.05$). Note that although the phenomenon of transsynaptic modulation of PTP is preserved, its degree is less prominent at near-physiological temperature than at room temperature, potentially because dendritic APs are shorter or glutamate spillover is less pronounced (Asztely et al., 1997). Data in (b, c) and (g, h, red bar) are from 9 pairs, data in (e, f) and (g, h, blue bar) are from 7 pairs. Error bars indicate SEM.

Supplementary Figure 3 | Pool analysis of PTP in Ca^{2+} channel blockers and mGluR antagonists, as in Fig. 3j–l and Fig. 4d–f.

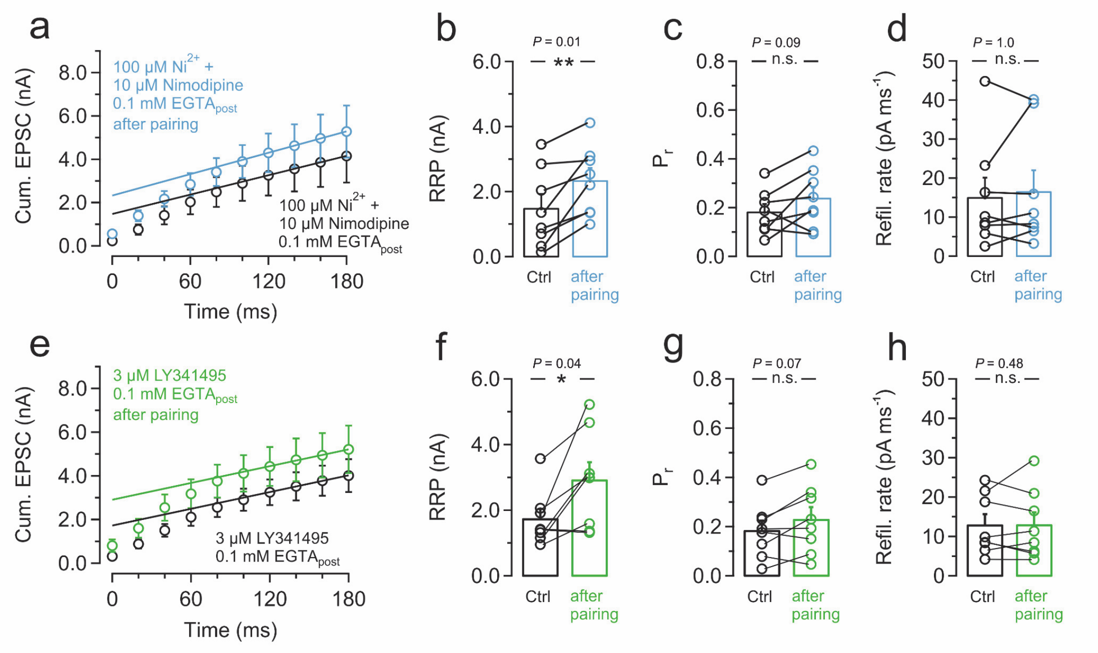

a–d, Pool analysis with 100 μM Ni^{2+} and 10 μM nimodipine in the bath and 0.1 mM EGTA in the postsynaptic pipette.

e–h, Pool analysis with 3 μM LY341495 in the bath and 0.1 mM EGTA in the postsynaptic pipette.

Last 4 data points were fit by linear regression, revealing readily releasable pool (RRP), release probability (P_r), and refilling rate. * indicates $P < 0.05$, ** $P < 0.01$, and n.s. denotes non-significant difference ($P \geq 0.05$). Data in (a–d) are from 8 pairs, data in (e–h) are from 8 pairs. Error bars indicate SEM.

Supplementary Figure 4 | Summary of effects of all pharmacological manipulations on the magnitude of PTP.

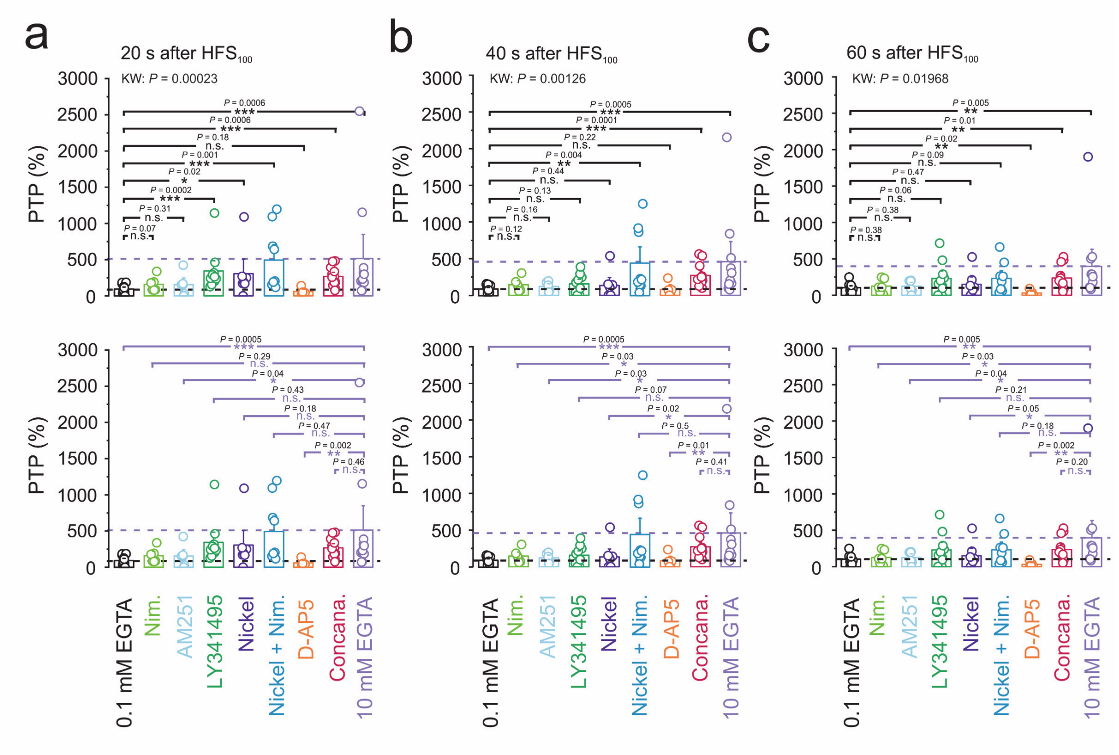

a, Comparison of PTP 20 s after HFS_{100} in the presence of various pharmacological manipulations with 0.1 mM EGTA (top) and 10 mM EGTA (bottom) in the postsynaptic pipette (representing the upper and lower ceiling of the magnitude of PTP).

b, PTP 40 s after HFS_{100}.

c, PTP 60 s after HFS_{100}.

In all mossy fiber terminal–CA3 pyramidal neuron pairs except those shown in the rightmost bar ("EGTA 10 mM"), the postsynaptic pipette solution contained 0.1 mM EGTA. In all pairs, the postsynaptic neuron was held in the current-clamp configuration during PTP induction. In 75 pairs, 100 stimuli were applied to the postsynaptic cells during PTP induction (CC_{100APs}). In 28 pairs, the postsynaptic cell was freely spiking during induction (CC_{free}). As results with CC_{100APs} and CC_{free} were not significantly different (Fig. 2g, two leftmost bars), data were pooled. Boxes represent mean values, and circles show individual measurements. Horizontal dashed lines represent 0.1 mM EGTA (bottom line, left bar) and 10 mM EGTA (top line, right bar). Differences were tested for statistical significance using a one-sided

Kruskal-Wallis test (χ^2 distribution with 9 – 1 degrees of freedom), followed by two-sided Mann-Whitney U tests. * indicates $P < 0.05$, ** $P < 0.01$, *** $P < 0.001$, and n.s. denotes non-significant difference ($P \geq 0.05$). P values are given without multiple comparison correction. Note that PTP in the presence of LY341495, Ca^{2+} channel blockers, and concanamycin was significantly different from 0.1 mM EGTA ($P < 0.05$ except LY341495 40 and 60 s after HFS_{100} and Ca^{2+} channel blockers 60 s after HFS_{100}), but not significantly different from 10 mM EGTA ($P \geq 0.2$ except LY341495 40 s after HFS_{100} and Ca^{2+} channel blockers 60 s after HFS_{100}), suggesting that the blockers largely recover PTP. Also note that the effects of pharmacological manipulations become less clear at later time points after PTP induction. Data are from 13, 6, 6, 10, 7, 9, 5, 12, and 11 pairs, respectively. Error bars indicate SEM.

Supplementary Reference

Asztely, F., Erdemli, G. & Kullmann, D. M. Extrasynaptic glutamate spillover in the hippocampus: dependence on temperature and the role of active glutamate uptake. *Neuron* **18**, 281–293 (1997).
